# Rab22a Promotes Epithelial-Mesenchymal Transition in Papillary Thyroid Carcinoma by Activating PI3K/AKT/mTOR Signaling Pathway

**DOI:** 10.1155/2022/1874550

**Published:** 2022-06-15

**Authors:** Xue Luo, Jinping Wang, Jinxi Lu, Xi Wang, Yuan Miao, Qingchang Li, Xiaoman Li, Liang Wang

**Affiliations:** ^1^Department of Pathology, The First Affiliated Hospital and College of Basic Medical Sciences, China Medical University, Shenyang, China; ^2^Key Laboratory of Medical Cell Biology, Ministry of Education, China Medical University, Shenyang, China

## Abstract

**Background:**

Rab22a is a member of the RAS superfamily, involved in early endosome formation and intracellular vesicle transport. Rab22a is significantly upregulated in a variety of malignant tumors. However, its function in thyroid cancer has never been addressed.

**Methods:**

The expression of Rab22a in paraffin sections of 101 patients was detected by immunohistochemical staining. By upregulating and downregulating the expression of Rab22a in thyroid cancer cell lines, the effect of Rab22a on cell proliferation, invasion, and migration was analyzed. Co-IP was employed, and the interaction between Rab22a and PI3Kp85*α* was shown. The function of Rab22a on PI3K/AKT/mTOR signaling and epithelial-mesenchymal transition (EMT) was further studied by western blot analysis.

**Results:**

Immunostaining showed that Rab22a was significantly overexpressed in thyroid cancer tissues but negative in adjacent normal tissues or nodular goiters. The proliferation, migration, invasion, and EMT in papillary thyroid carcinoma cell lines were enhanced upon Rab22a overexpression but inhibited after knocking down Rab22a. The co-IP assay demonstrated an interaction between Rab22a and PI3K85*α*, an effector of PI3K. We further found that Rab22a can activate the PI3K/AKT/mTOR signaling pathway. However, the ability of Rab22a to promote the proliferation, invasion, migration, and EMT of papillary thyroid carcinoma cells was significantly inhibited after being treated with LY294002, a PI3K inhibitor.

**Conclusions:**

Rab22a can promote the EMT process and enhance proliferation, migration, and invasion of papillary thyroid carcinoma cells by activating the PI3K/AKT/mTOR signaling pathway. Our study provides new pathological diagnosis clues and clinical treatment targets for thyroid cancer.

## 1. Introduction

Thyroid cancer is one of the most common endocrine malignant tumors, especially among young women, and its incidence has been increasing rapidly in recent decades worldwide [1]. Among the malignant tumors affecting women, its incidence ranks fifth in the world [2]. Most thyroid cancers are slow-growing tumors, and the vast majority of cancer patients have a good prognosis. However, patients with relapsed or undifferentiated thyroid cancer often have a poor prognosis [3]. Previous studies have shown that abnormalities in some target genes and related signal pathways are closely associated with the occurrence and development of thyroid cancer [4, 5].

Rab proteins are members of small GTPases in the Ras superfamily, and most Rabs are the key regulators of intracellular membrane transport. The first Rab protein was discovered in 1983. Until now, about 70 different Rabs have been discovered [6]. The Rab family is closely related to cell metabolism, autophagy, and signal transduction and may further promote the progression of tumors [7]. Rab22 protein has two subtypes, Rab22a and Rab22b, which are widely expressed in mammalian tissues [8]. Rab22a was initially found in dogs and had the highest similarity with Rab5 homologous sequences [9]. Rab22a is associated with primary and secondary endosomes, but not lysosomes. It mediates the transformation of primary endosomes to secondary endosomes and plays a key role in the endocytic pathway [10]. In addition, many studies have confirmed that Rab22a is upregulated and closely related to the progress of various malignant tumors [11–15]. However, the function of Rab22a in thyroid malignancy has never been studied.

The PI3K/AKT/mTOR signaling pathway has been widely studied and considered a pathway involved in regulating cell cycles [16], proliferation, and survival [17], as well as cell metabolism and autophagy [18]. Previous studies have demonstrated that, in thyroid tumors, multiple oncogenes were closely related to this signaling pathway. Santoro et al. reported that *RET*/*PTC* chromosomal rearrangement activated the PI3K/AKT pathway in papillary thyroid carcinoma [19]. Miller et al. revealed that PI3K signaling induced by *KRAS* and activation of *ERK* could induce the transformation of thyroid epithelial cells [20]. Moreover, PTEN may negatively regulate the PI3K/PKB/AKT signaling pathway to exert its tumor suppressor effect [21]. Many related studies have linked this pathway to the pathogenesis of thyroid tumors. However, the relationship between Rab22a and the PI3K/AKT/mTOR signaling pathway in thyroid cancer has never been explored.

In this study, we compared the expression of Rab22a in thyroid cancer tissues and nodular goiters. By bi-bidirectionally manipulating the expression of Rab22a, its function in proliferation, migration, invasion, and epithelial-mesenchymal transition (EMT) was investigated in papillary thyroid carcinoma cells. We further demonstrated the tumor-promoting effect of Rab22a with the PI3K/AKT/mTOR signaling pathway.

## 2. Materials and Methods

### 2.1. Patients and Analysis of Clinicopathological Parameters

Thyroid tissue specimens (31 papillary thyroid carcinomas, 33 follicular thyroid carcinomas, 7 medullary thyroid carcinomas, and 30 nodular goiters) were collected from patients who underwent surgical treatment at the First Affiliated Hospital of China Medical University between 2019 and 2020. Histopathological diagnosis and grading were performed according to the 2017 World Health Organization (WHO) classification of thyroid tumors. Clinical tumor staging was analyzed according to the eighth edition of the American Joint Committee on Cancer (AJCC) thyroid cancer staging system. The local institutional review committee approved our research at the China Medical University. A retrospective analysis was performed on the primary data of patients and related clinicopathological parameters, such as sex, age, tumor size, lymph node metastasis, and clinical stage. A retrospective analysis of the primary data of patients was performed, and a statistical analysis of relevant clinicopathological parameters such as gender, age, tumor size, *BRAFV600E* mutation, lymph node metastasis, and clinical staging was performed.

### 2.2. Immunochemical Staining and Evaluation

Thyroid tumor specimens were processed through specific procedures. First, they were fixed with 10% neutral formalin, then embedded in paraffin, and finally cut into 4 *μ*m thick serial sections for the following related experiments. Immunohistochemical staining was performed using the streptavidin-peroxidase method. After deparaffinization of thyroid tissue sections, citrate is used for antigen retrieval under high temperature and high-pressure conditions. The sections were incubated with anti-Rab22a rabbit polyclonal antibody (1 : 500, HPA066920, Sigma-Aldrich, Shanghai, China) at 4°C overnight. Next, incubate with biotinylated goat anti-rabbit IgG secondary antibody. After washing, the tissue sections were cultured with horseradish peroxidase-conjugated streptavidin-biotin (Ultrasensitive; Maixin, Fuzhou, China). 3,3-Diaminobenzidine tetrahydrochloride (Maixin) was used for staining according to the product manual. Tissue sections were stained with hematoxylin and mounted for evaluation. Two professional pathologists blinded to the relevant clinical data evaluated the tissue staining intensity and the percentage of stained cells in the specific area and semiquantitatively scored the tissue sections. The detailed semiquantitative scoring standards were as follows: 0 indicated no staining, 1 weak staining, 2 moderate staining, and 3 strong staining, and the percentage of positive cells was scored as 0 (negative), 1 (1-25%), 2 (26-50%), 3 (51-75%), or 4 (76-100%). By correlating the intensity and percentage scores, a final score of 0 to 12 was produced. A score ≥ 4 was considered high expression, whereas a score < 4 was defined as low expression.

### 2.3. Cell Culture and Plasmid Transfection

The human papillary thyroid carcinoma cell lines TPC1 and K1 were obtained from the Shanghai Cell Bank (Shanghai, China) and cultured in RPMI-1640 medium (HyClone, South Logan, UT, USA) with 10% fetal bovine serum (Gibco, Rockville, MD, USA), 100 *μ*/mL penicillin, and 0.1 mg/mL streptomycin. At 70% confluence, the cells were transfected with the described plasmids or siRNAs using Lipofectamine 3000 (Invitrogen, Carlsbad, CA, USA) according to the manufacturer's instructions. 48 hours later, transfected cells were collected for subsequent experiments. The Rab22aWT (wild-type), Rab22a-S19N, and Rab22a-Q64L plasmids were generous gifts from Dr. Guangpu Li (University of Oklahoma Health Sciences Center, OK, USA). Rab22a siRNA was purchased from General Biosystems (Anhui, China). Scrambled siRNAs were used as the negative control, and the base sequences were 5′-UUCUCCGAACGUGUCACGUTT-3′ and 5′-ACGUGACACGUCGGAGAATT-3′.

### 2.4. Western Blot

TPC1 and K1 cells transfected with different plasmids were collected 48 hours later. Total protein was extracted using cell lysate buffer prepared with NP40, PMSF, and protease inhibitors. Protein samples were separated with 10% sodium dodecyl sulfate-polyacrylamide gel electrophoresis (SDS-PAGE) (Beyotime, Shanghai, China). Protein was transferred to a polyvinylidene fluoride (PVDF) membrane (Millipore, Billerica, MA, USA) at 140 mA for 90 min, and the membrane was blocked in 5% skim milk for 2 h. Primary antibodies were incubated at 4°C overnight and incubated with secondary antibodies for 1 h. The bands were exposed by electrochemiluminescence (ECL) and analyzed using ImageJ software (National Institute of Health, USA). The information on the relevant antibodies is provided in Table S1.

### 2.5. Cell Counting Kit-8 Assay

TPC1 and K1 cells were plated in 96-well plates in the medium containing 10% FBS at 3000 cells per well 48 h after transfection. The cells were stained with Cell Counting Kit-8 (CCK8, Apexbio) at the same time on days 1 to 5. The absorbance at 450 nm was measured to determine the cell proliferation rate.

### 2.6. Colony Formation Assay

Forty-eight hours after transfection, the cells were plated in 6 cm cell culture dishes at 300 cells per dish with a medium refresh every 3 days. A week later, the cells were first fixed with 4% paraformaldehyde for 20 minutes and then stained with 0.1% crystal violet for 15 minutes. Colonies > 0.3 mm in diameter were counted and recorded.

### 2.7. Transwell Cell Migration and Invasion Assay

The cells were plated in a serum-free medium at a concentration of 2.0 × 10/mL. A Transwell chamber with or without Matrigel precoating was inserted into a 24-well plate with 200 *μ*L of suspension in the apical chamber and 600 *μ*L of medium with 20% FBS in the basolateral chamber. After 24 h, the chambers were removed, and the cells that penetrated the membrane were fixed with 4% paraformaldehyde for 20 min and subsequently stained with 0.1% crystal violet for 15 min. For each membrane, 5 randomly selected fields were captured for counting (magnification, 20×). Transwell chambers for the invasion assay were precoated with Matrigel.

### 2.8. Wound Healing Assay

When the cells reached 70% confluency in a 6-well plate, the cells were transfected with the stated plasmids. After the cells were 100% confluent, a 200 *μ*L pipette tip perpendicular to the bottom of the plate was used to produce a straight scratch. The movement of the cells into the wound was assessed by imaging the scratch in the same position (0 h and 24 h) with an inverted microscope. The images were analyzed by ImageJ, and the cell migration rate was calculated and measured.

### 2.9. Co-Immunoprecipitation (Co-IP)

TPC1 and K1 cells were transfected with Rab22a plasmid and collected after 48 hours. Cell lysis buffer consisting of NP40, PMSF, and protease inhibitor was added, lysed on ice for 30 min, and centrifuged to get the supernatant. A small amount of supernatant was taken for subsequent western blot analysis. The remaining lysate is divided into two parts. Appropriate amounts of IgG (Cell Signaling Technology) antibody and Rab22a antibody were added, respectively, and placed in a 4°C shaker incubate overnight. Protein A+G agarose (Beyotime) was added to the lysate after antibody incubation overnight and was incubated in a shaker at 4°C for 2-4 hours to couple the antibody to Protein A+G agarose. Then, a centrifuge was performed at 3,000 rpm at 4°C for 3 minutes to collect precipitation. The precipitated sample was washed and subjected to western blot analysis.

### 2.10. Statistical Analysis

All statistical analyses involved in the experiments used SPSS version 22.0 for Windows (SPSS, Chicago, IL, USA). Pearson's chi-square test and Fisher's test were used to evaluate the possible relationship between Rab22a and clinicopathological factors. The paired *t*-test was used to analyze CCK8, colony formation, wound healing, and Matrigel invasion assay data. *P* < 0.05 denotes statistical significance.

## 3. Results

### 3.1. Rab22a Is Overexpressed in Thyroid Malignancies

**Figure 1:**
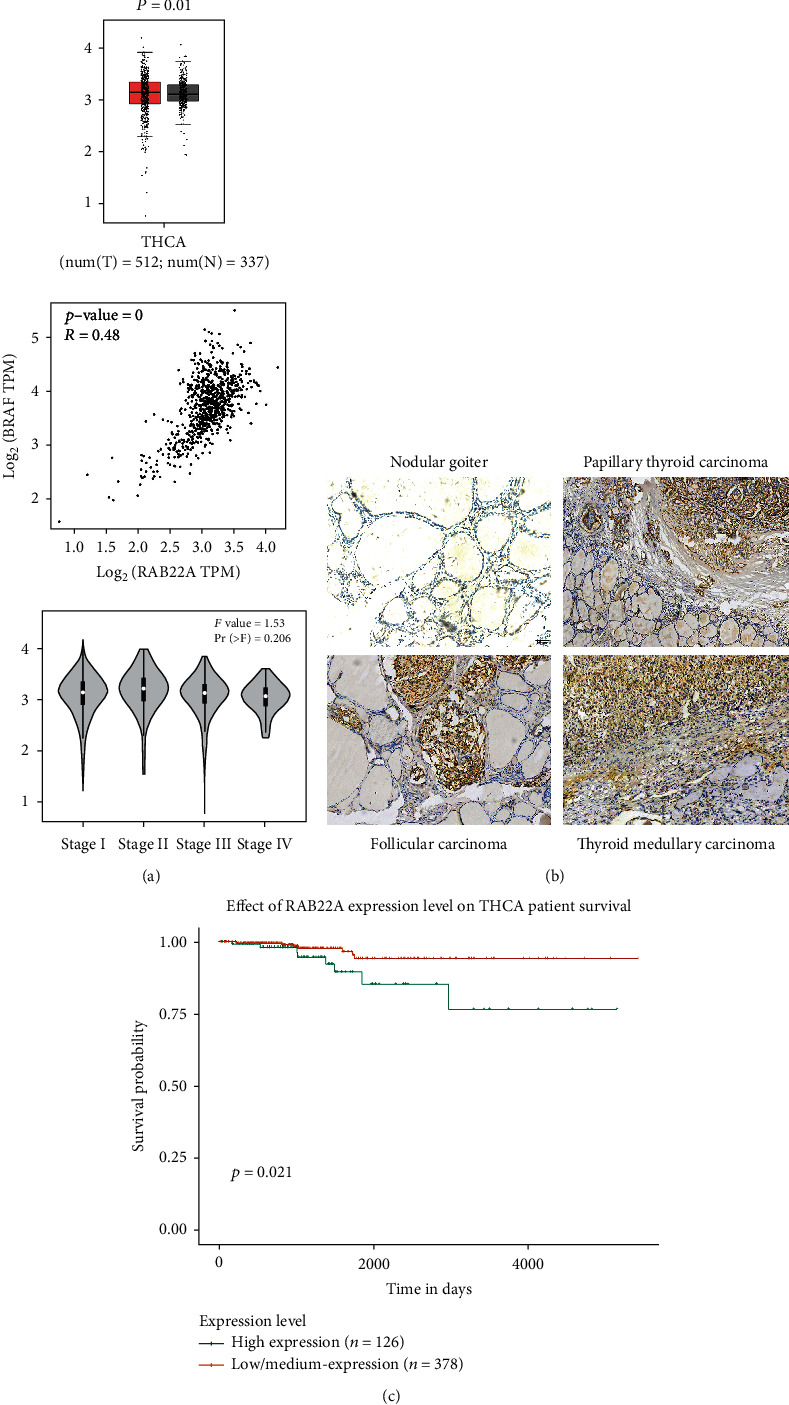
Rab22a is overexpressed in thyroid malignancies. GEPIA database showed the relationship between the expression of *RAB22A* with tumors (*P* = 0.01), *BRAF* mutations (*P* = 0), and stages (Pr = 0.206) (a). Representative images of Rab22a protein expression in papillary thyroid carcinoma, thyroid follicular carcinoma, medullary thyroid carcinoma, and nodular goiter tissues (b, 100×). UALCAN analysis showed that the patients with high *RAB22A* expression had poorer overall survival (c).

To investigate the correlation between Rab22a and thyroid tumor tissues, UALCAN and GEPIA online databases were used to analyze the expression of Rab22a in normal thyroid tissues and tumors. We found that the expression of *RAB22A* in thyroid tumor tissues was higher than that in normal tissues. Meanwhile, the expression of *RAB22A* is positively correlated with *BRAF* gene mutations. In addition, database analysis shows that the expression of *RAB22A* in different stages of thyroid tumors has no obvious difference (Figure 1(a)) (GEPIA (cancer-pku.cn)). The survival analysis curve shows that the expression of *RAB22A* is negatively correlated with patient survival, indicating that it is associated with poor prognosis (Figure 1(c)) (http://ualcan.path.uab.edu/analysis). Next, we collected the pathological tissue samples of 101 patients who underwent complete surgical resection in the First Affiliated Hospital of China Medical University from 2019 to 2020, and immunohistochemical staining was performed. In papillary thyroid carcinoma, thyroid follicular carcinoma, and medullary thyroid carcinoma, Rab22a was positively overexpressed in tumor tissues and negatively expressed in adjacent normal thyroid tissues or nodular goiters (Figure 1(b)). Statistical analysis of the expression of Rab22a and related clinicopathological parameters revealed that the expression of Rab22a in benign thyroid tissues and malignant thyroid tumors has significant differences. However, no obvious differences were found among the three types of malignant tumors, and there was no significant correlation with other factors. The detailed clinicopathological parameter analysis is shown in Table 1.

### 3.2. Rab22a Promotes Malignant Biological Behaviors in Thyroid Cancer Cells

**Figure 2:**
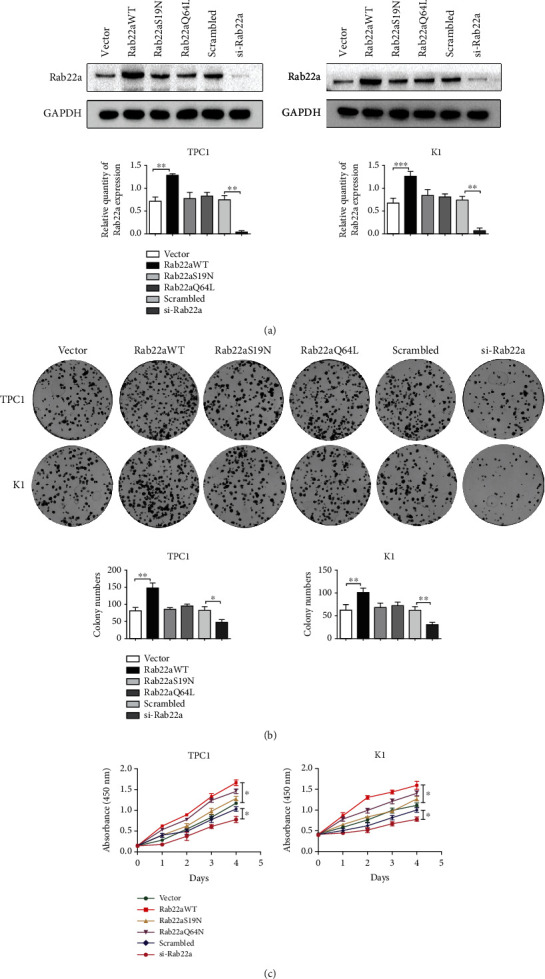
Rab22a promotes malignant biological behaviors in thyroid cancer cells. The level of Rab22a was increased or decreased after transfection with different plasmids or specific interference plasmids of Rab22a, respectively (a). Rab22a promoted the proliferation of TPC1 and K1 cells, as shown by colony formation and CCK8 assays (b and c). Statistical data were the mean ± SD of the three repeated independent trials: ^∗∗∗^*P* < 0.001, ^∗∗^*P* < 0.01, and ^∗^*P* < 0.05.

**Figure 3:**
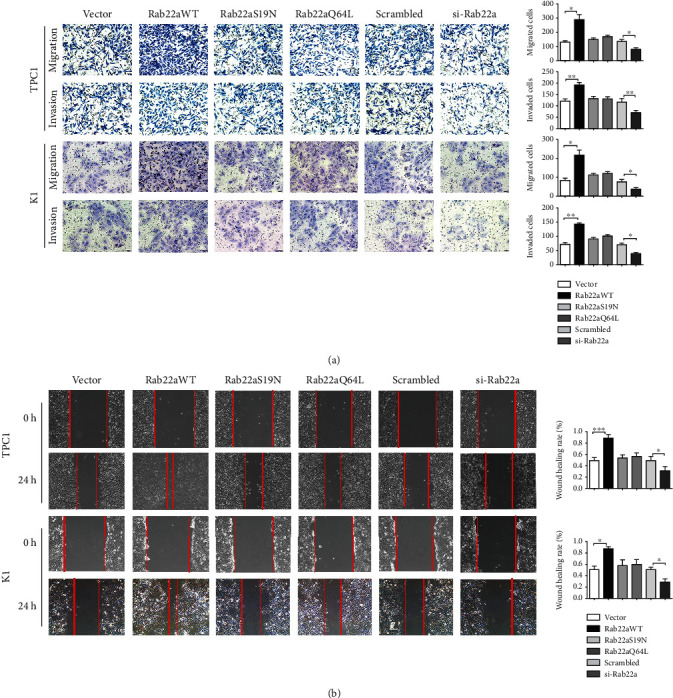
Rab22a promotes the migration and invasion of thyroid cancer cells. Transwell assays showed that Rab22aWT increased the migration and invasion abilities of TPC1 and K1 cells, and Rab22a knockdown showed the opposite effect (a, 200×). The migration ability of TPC1 and K1 cells was assessed by wound healing assays (b, 200×). Statistical data were the mean ± SD of the three repeated independent trials: ^∗∗∗^*P* < 0.001, ^∗∗^*P* < 0.01, and ^∗^*P* < 0.05.

To further explore the correlation between Rab22a and thyroid tumors, we conducted a series of in vitro experiments in thyroid papillary carcinoma cell lines, TPC1, and K1, to detect the effect influence of Rab22a on malignant behavior of cells. We transfected Rab22aWT, Rab22aS19N (GTPase dominant-negative mutant), and Rab22aQ64L (GTPase dominant-active mutant) plasmids and Rab22a-specific siRNA into TPC1 and K1 cells, respectively. Meanwhile, vector and scrambled siRNA are used as the control group. The expression of Rab22a was confirmed by western blot (Figure 2(a)). The colony formation experiment and CCK8 test showed that Rab22aWT could promote the proliferation of TPC1 and K1 cells, while there was no significant difference in Rab22aS19N or Rab22aQ64L. On the contrary, when in cells transfected with Rab22a-specific siRNA, the proliferation ability of the cells was inhibited compared with the scrambled siRNA group (Figures 2(b) and 2(c)). Transwell analysis showed that, upon transfection of Rab22aWT, both migration and invasion were enhanced in thyroid cancer cells, compared with the control group. However, the cell migration and invasion, as demonstrated by the Transwell and wounding healing assay, were inhibited when Rab22a was knocked down. The Rab22a mutants have no obvious effect. (Figure 3(a)). In the wound healing assay, consistent results were obtained (Figure 3(b)). In summary, the overexpression of Rab22a was positively correlated with the proliferation, invasion, and migration of cells.

### 3.3. The Effect of Rab22a on PI3K/AKT/mTOR Signaling Pathway

**Figure 4:**
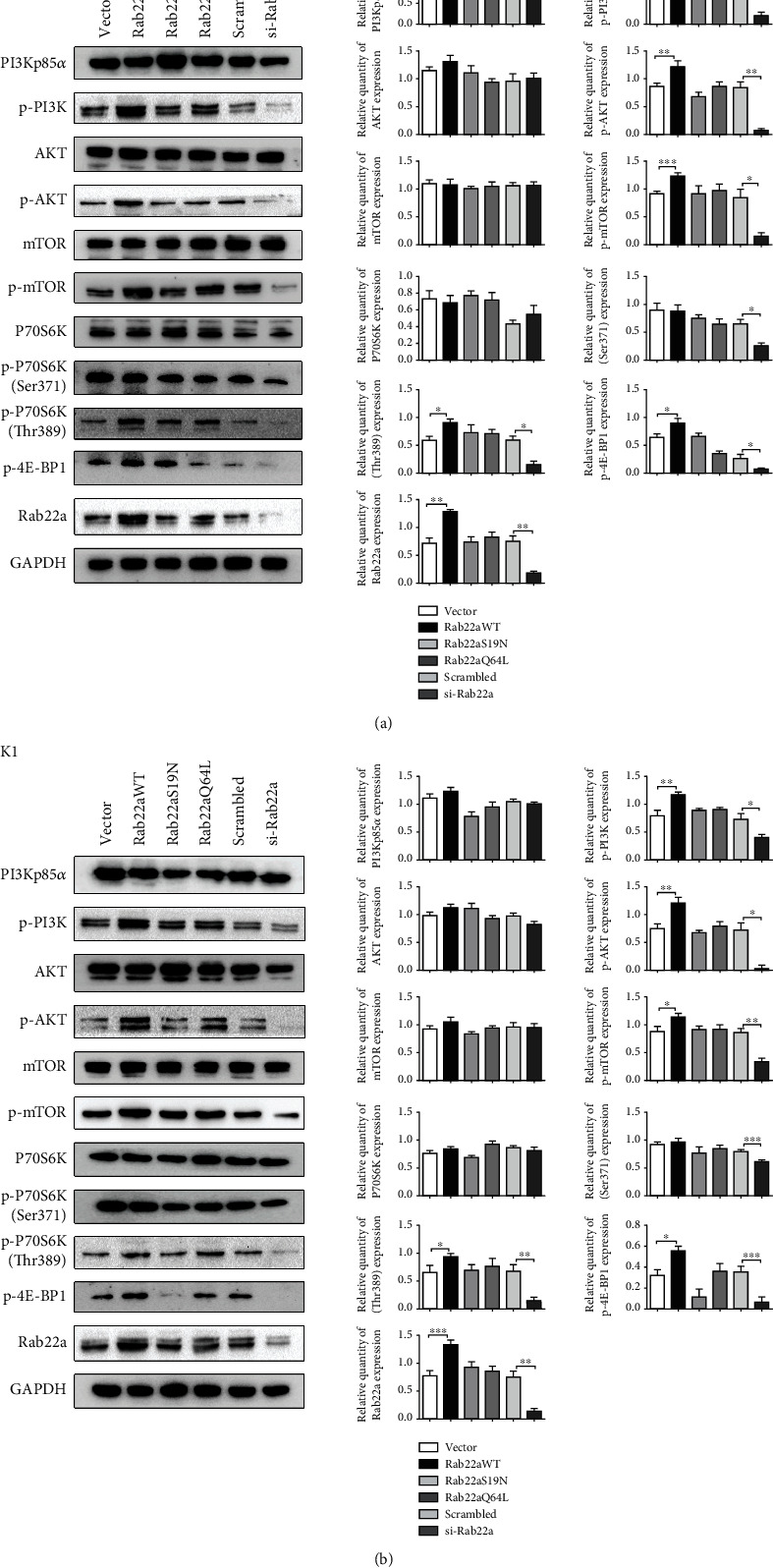
The effect of Rab22a on PI3K/AKT/mTOR signaling pathway. Western blot analysis of the expression of PI3Kp85*α*, p-PI3K, AKT, p-AKT (Ser473), mTOR, p-mTOR (Ser2448), p70S6K, p-p70S6K (Ser371), p-p70S6K (Thr389), p-4E-BP1 (Thr37/46), and Rab22a in TPC1 and K1 cells (a and b). Statistical data were the mean ± SD of the three repeated independent trials: ^∗∗∗^*P* < 0.001, ^∗∗^*P* < 0.01, and ^∗^*P* < 0.05.

It has been previously reported that the progression of thyroid cancer is closely related to the activation of the PI3K/AKT signaling pathway [22–24]. Rab5, a Rab with nearly 50% amino acid sequence identity with Rab22a, can interact with PI3K core regulatory subunit p85*α* [25]. Therefore, we examined the relationship between Rab22a and PI3Kp85*α* by Co-IP experiment in both TPC1 and K1 cell lines and verified the interaction between Rab22a and PI3Kp85*α* (Figure S1). We further explored the influence of Rab22a on the proteins related to the PI3K/AKT/mTOR signaling pathway by western blot. The results showed that, after transfection with Rab22aWT, the expression of PI3K/AKT/mTOR signaling pathway-related proteins, such as p-PI3K, p-AKT (Ser473), p-mTOR (Ser2448), p-p70S6K (Thr389), and p-4E-BP1 (Thr37/46), was increased, whereas the expression levels of related phosphorylated proteins decreased by knocking down Rab22a. Meanwhile, no significant differences were identified in the groups of Rab22a mutants (Figures 4(a) and 4(b)).

### 3.4. Rab22a Promotes the Proliferation, Invasion, and Migration of Thyroid Cancer Cells by Regulating the PI3K/AKT/mTOR Signaling Pathway

**Figure 5:**
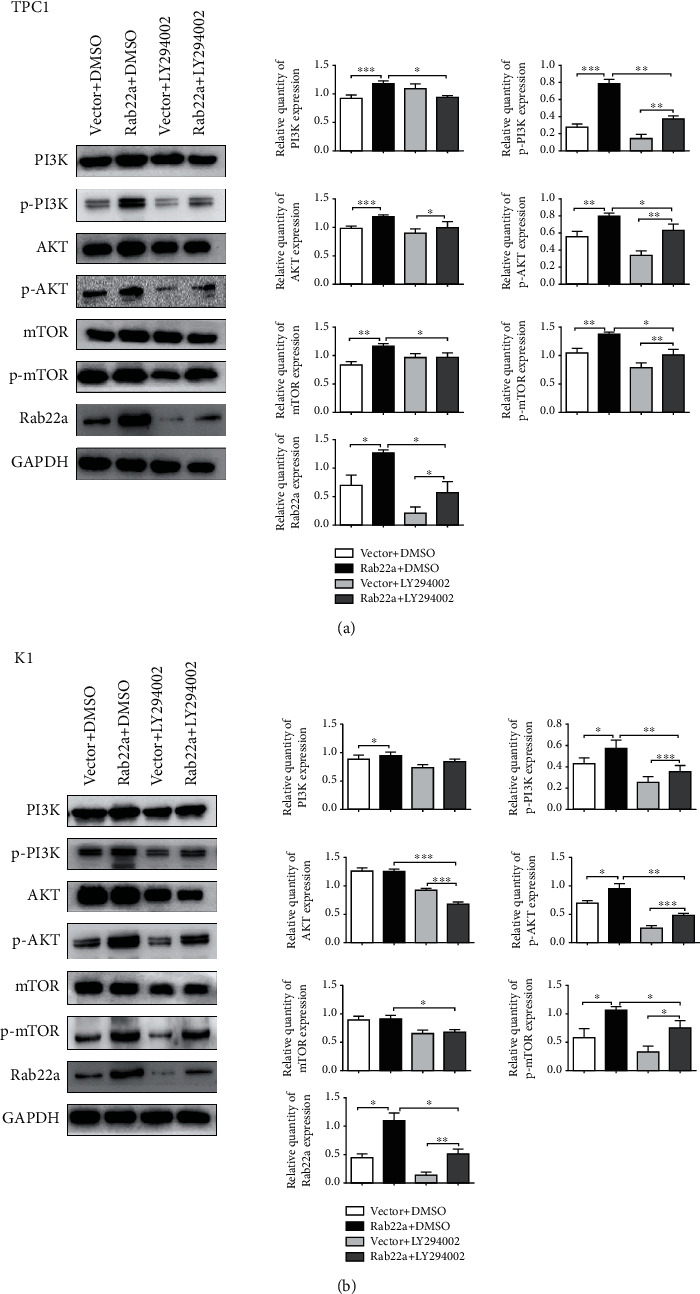
The influence of Rab22a on PI3K/AKT/mTOR signaling pathway. The western blot analysis results revealed that the expression of p-PI3K, p-AKT (Ser473), p-mTOR (Ser2448), and Rab22a induced by Rab22a overexpression or decreased by PI3K inhibitors in TPC1 and K1 (a and b). Statistical data were the mean ± SD of the three repeated independent trials: ^∗∗∗^*P* < 0.001, ^∗∗^*P* < 0.01, and ^∗^*P* < 0.05.

**Figure 6:**
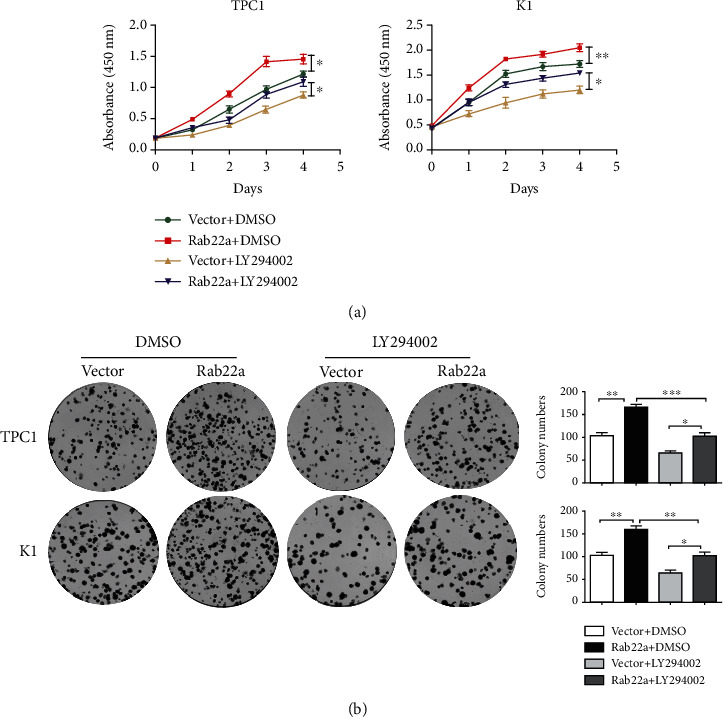
The PI3K inhibitors reduced Rab22a promoting the proliferation of thyroid cancer cells. The TPC1 and K1 cell proliferation were decreased by PI3K inhibitor treatment, as shown by CCK8 and colony formation assays (a and b). Data are shown as mean ± SD of the three independent experiments: ^∗∗∗^*P* < 0.001, ^∗∗^*P* < 0.01, and ^∗^*P* < 0.05.

**Figure 7:**
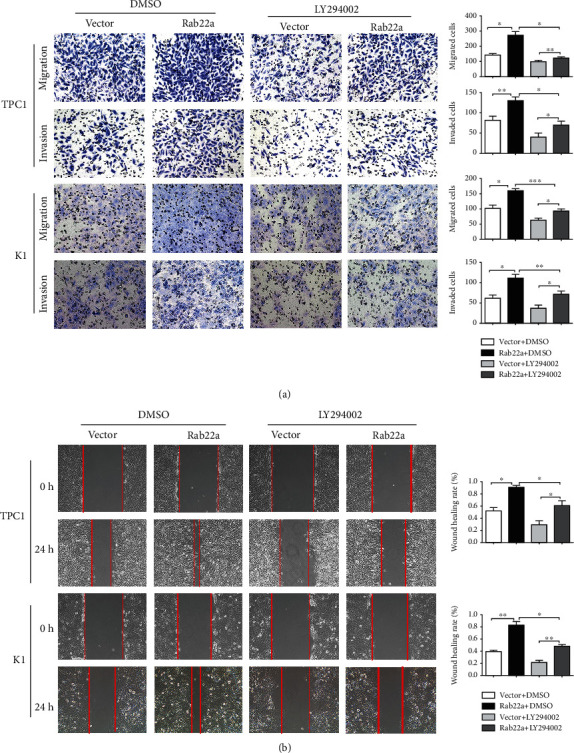
The Rab22 promoted invasion and migration of thyroid cancer cells was weakened by PI3K inhibitors. Transwell assays showed that the migration and invasion of TPC1 and K1 cells decreased by PI3K inhibitors (a, 200×). The migration of TPC1 and K1 cells was assessed by wound healing assays (b, 200×). Statistical data were the mean ± SD of the three repeated independent trials: ^∗∗∗^*P* < 0.001, ^∗∗^*P* < 0.01, and ^∗^*P* < 0.05.

As an inhibitor of PI3K, LY294002 has been widely used and can significantly inhibit the expression of PI3K/AKT/mTOR-related signaling pathway proteins [26–28]. To further study the targeted regulation relationship between Rab22a and PI3K/AKT/mTOR signaling pathway, we treated Rab22a-transfected TPC1 and K1 cells with LY294002, using DMSO as the control. Through preexperiment and exploration of the concentration gradient, the inhibitory effect was the most obvious at 50 *μ*m (Figure S2). Meanwhile, we observed the expression of proteins related to the PI3K/AKT/mTOR signaling pathway at this concentration, and the results showed that, in TPC1 and K1 cell lines, the expression levels of p-PI3K, p-AKT (Ser473), p-mTOR (Ser2448), and other related proteins were all decreased compared with the vector group (Figures 5(a) and 5(b)). Consistent results were observed in the colony formation test, CCK8 test (Figures 6(a) and 6(b)), Transwell assay, and wound healing assay (Figures 7(a) and 7(b)), indicating that LY294002 could attenuate the promoting effect of Rab22aWT on the proliferation, migration, and invasion of TPC1 and K1 cells. Altogether, these results suggest that Rab22a can enhance the malignant biological behavior of thyroid cancer cells by activating the PI3K/AKT/mTOR signaling pathway.

### 3.5. Rab22a Promotes the EMT Process of Thyroid Cancer Cells by Regulating PI3K/AKT/mTOR Signaling Pathway

**Figure 8:**
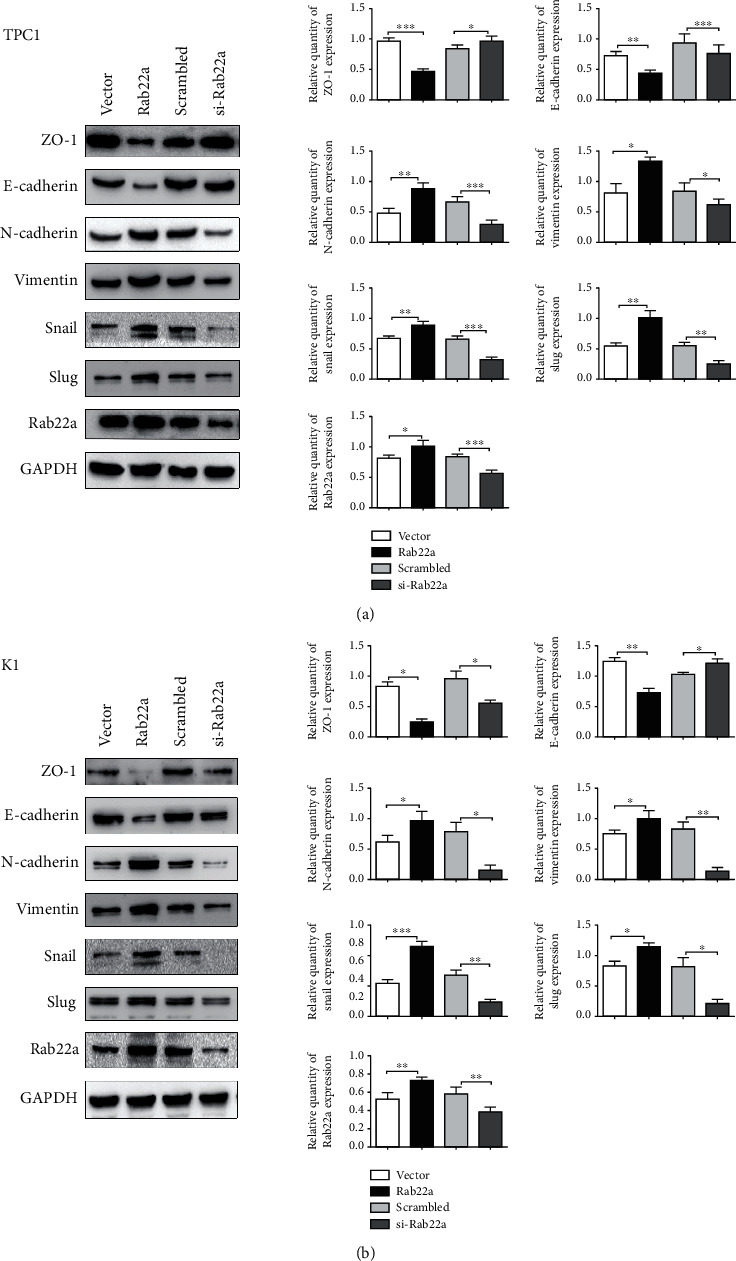
Rab22a promotes the EMT process of thyroid cancer cells. The western blot analysis results revealed the expression of EMT-related proteins induced by Rab22a overexpression or knockdown in TPC1 (a) and K1 cells (b). Statistical data were the mean ± SD of the three repeated independent trials, ∗∗∗*P* < 0.001, ∗∗*P* < 0.01, and ∗*P* < 0.05.

**Figure 9:**
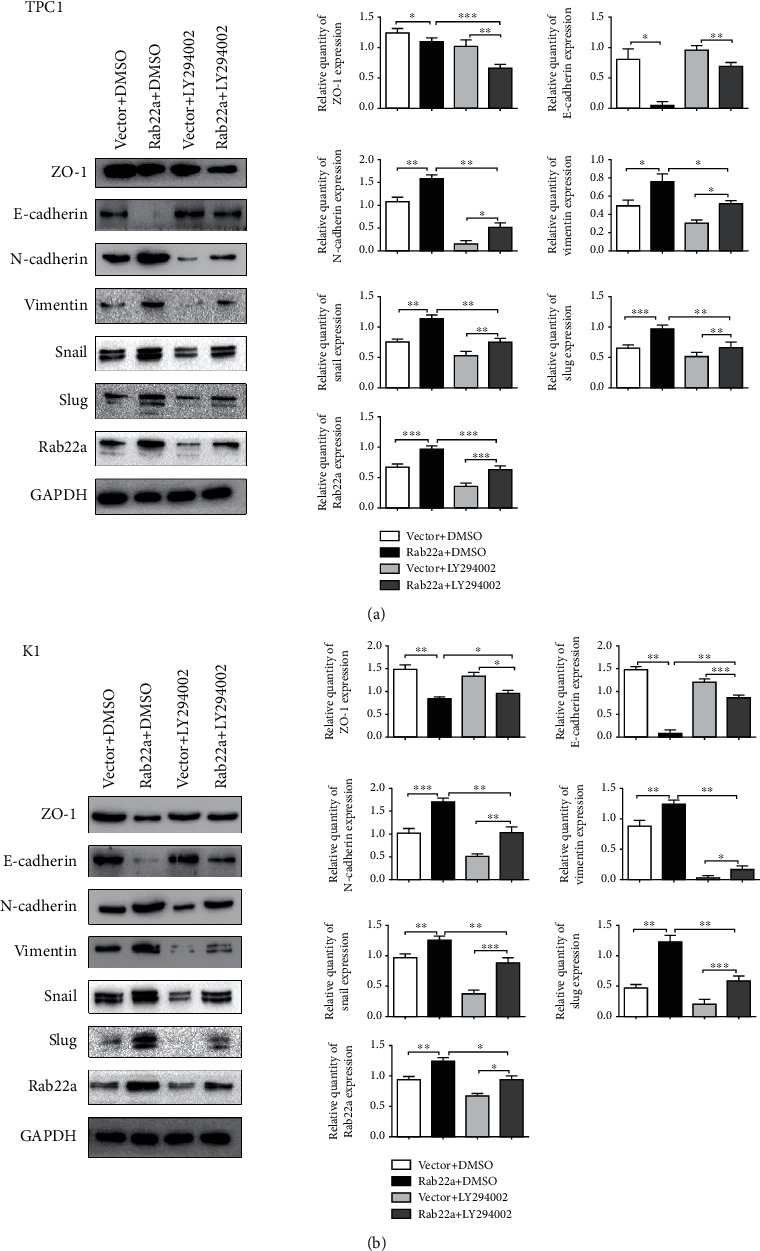
Rab22a regulated the EMT process of thyroid cancer cells via PI3K/AKT/mTOR signaling pathway. Western blot analysis showed that the expression of EMT-related proteins upon Rab22a transfection was changed after treatment with PI3K inhibitors (a, b). Statistical data were the mean ± SD of the three repeated independent trials: ^∗∗∗^*P* < 0.001, ^∗∗^*P* < 0.01, and ^∗^*P* < 0.05.

The carcinogenesis and malignant progression of various malignant tumors are closely related to EMT, which can be regulated by PI3K/AKT/mTOR signaling pathway [29–32]. In this study, the expression of Rab22a is positively correlated with EMT-related proteins, such as N-cadherin, vimentin, Snail, and Slug (Figures 8(a) and 8(b)). Next, to further verify the correlation between EMT and Rab22a, we conducted a parallel assay in Rab22a-overexpressed cells, treated with LY294002 or DMSO. Compared with the DMSO group, the EMT-promoting effect was repressed upon LY294002 treatment (Figures 9(a) and 9(b)). Taken together, these results suggested that, in thyroid cancer cells, Rab22a may promote EMT through upregulating PI3K/AKT/mTOR signaling pathway.

## 4. Discussion

Most of the 60 known members of the Rab family have specific localization in specific membrane-binding loci, and perturbation of Rab function significantly alters the transport steps of multiple membranes. These little GTPases act as molecular “on/off” switches to cycle between inactive (GDP binding) and active (GTP binding) states [33]. Rab22a plays a joint role in endocytosis and recycling [34]. Colocalization studies have shown Rab22a exists in the early endosomes, interacting with EEA1 and participating in biosynthesis and early endocytic pathways [9]. In CHO cells, overexpression of Rab22a promotes fusion between endosomes and hinders transport from endosomes to the Golgi apparatus^[10; 35]^. Rab22a can regulate the endocytic cycle of CD147 [35]. By enhancing the recycling of CD147, Rab22a promotes the invasion and migration of lung cancer cells [36]. In this study, Rab22aWT has a promotion effect on the malignant progression of thyroid tumors. In contrast, GTP continuous binding mutants (Rab22aQ64L) and GDP continuous binding mutants (Rab22aS19N) have no significant effect on the development of thyroid tumors. Rab22aWT and mutant have different functions in endocytosis, which have different effects on the morphology and function of endosomes [10]. Overexpression of the GDP binding mutants can disrupt the TGN positioning of TGN46 and play a role in the antegrade exit of TGN, while the Rab22aWT does not participate in this pathway [7]. The differences in the endocytic role of different Rab22 mutants will largely affect the differences in protein expression levels. Rab22a was reported as an oncogene in several previous works of the literature. It has been reported that SNHG3 promotes the overexpression of Rab22a by regulating miRNA-151A3p in osteosarcoma, thereby promoting the invasion and migration potentials of osteosarcoma cells [15]. He et al. revealed that Rab22a was associated with poor prognosis in breast cancer patients by regulating miR-193b, and the downregulation of Rab22a significantly reduced the proliferation, migration, and invasion of breast cancer cells [13]. Rab22a is a direct downstream target of miR-520b, upregulated in gallbladder cancer, and negatively correlated with miR-520b expression. When the expression of Rab22a was inhibited, the proliferation, invasion, and migration ability of gallbladder cancer cells were significantly inhibited [11]. Xiong et al. confirmed that overexpression of Rab22a promoted the proliferation and invasion of renal cell carcinoma, and this behavior may be related to the direct targeting of miR-204 to *RAB22A* [12]. In the current study, we found that the expression of Rab22a was upregulated in thyroid cancer. Overexpression of Rab22aWT can significantly enhance the proliferation, migration, and invasion of thyroid cancer cells, TPC1, and K1 cells. However, the proliferation, migration, and invasion were significantly inhibited when Rab22aWT was downregulated. We further investigated the influence of Rab22a on the PI3K/AKT/mTOR signaling pathway. First, by Co-IP, we confirmed that Rab22a can interact with PI3Kp85*α*. Second, we found that the key effectors of the PI3K/AKT/mTOR signaling pathway were upregulated upon overexpression of Rab22a WT, while knocking down Rab22a resulted in the opposite effect. In addition, the enhancement of Rab22a can be reversed by the treatment of LY294002, the PI3K inhibitor.

The results of our research are consistent with the previous reports that the overexpression of Rab22a is associated with tumor malignancy. The current study further explored the relationship between Rab22a and PI3K/AKT/mTOR signaling pathway to verify the influence of Rab22a on the malignant progression of thyroid cancer cells, which was unprecedented. Previous research has reported that the PI3K/AKT/mTOR signaling pathway may be associated with EMT and are closely related to the progression of various tumors. Yang et al. revealed that DEK promoted EMT and angiogenesis in triple-negative breast cancer by regulating the PI3K/AKT/mTOR signaling pathway [37]. As shown in the previous reports, CircEPSTI1 interacts with miR-942-5p and promotes EMT through phosphorylation of PI3K/AKT/mTOR, thereby promoting proliferation and invasion of oral squamous cell carcinoma (OSCC) cells [29]. Zhang et al. verify that circNRIP1 could successfully upregulate EMT markers by activating the AKT/mTOR signaling pathway and further promote the growth of gastric cancer cells in vivo [14]. Y. H. Yeh et al. confirmed that HIF-1*α* promoted tumor EMT and affected tumor progression by activating this critical signaling pathway [38]. Based on previous studies, we further studied the correlation between Rab22a and EMT. We found that, in TPC1 and K1 cells, the expression of E-cadherin and ZO-1 was downregulated after transfection of Rab22a, while the PI3K inhibitor reversed this trend. The protein levels of N-cadherin, vimentin, Snail, and Slug were significantly upregulated upon Rab22a overexpression, which can be inhibited upon LY294002 treatment. Rab22aWT promotes the EMT process of thyroid cancer. Together with the previous studies, it indicated that the EMT-promoting effect of Rab22 was conducted through upregulation of PI3K/AKT/mTOR signaling pathway. It needs further study whether Rab22a jointly affects the EMT process by regulating endocytosis and plasma membrane trafficking. In particular, if it can be verified through animal models or organoid models whether Rab22a really has the effect of promoting tumor cell invasion, metastasis, and enhancing EMT for papillary thyroid carcinoma and other histological subtypes *in vivo*, it will be more convincing and may also reveal more complex related mechanisms.

In our study, Rab22aWT significantly enhanced malignant biological behavior in thyroid cancer cells, while mutants (Rab22aQ64L and Rab22aS19N) had no such effect. Previous studies revealed that overexpression of Rab22aWT affected the transport between endosomes and the Golgi apparatus by promoting the fusion of endosomes and damaging the Golgi network^[9; 35]^. Mesa showed that Rab22aQ64L did not promote the fusion of all endocytic compartments in a kinetic model, and Rab22aS19N has no significant effects [39]. Rab22aS19N has reduced affinity for GTP and showed decreased endocytosis of a fluid phase marker [10]. Consistently, Rab22aS19N could inhibit clathrin-independent endocytosis, which can affect T cells [40]. We further speculated that the damage to the endocytic pathway might affect protein synthesis in cells. As shown in this study, compared with the Rab22aWT, the expression of the Rab22 mutants at the protein level is lower. The significant impact of Rab22aWT might be closely related to the aforementioned protein levels and functional cycle between GTP/GDP binding statuses. However, the specific mechanism needs to be further studied.

In summary, the thyroid cancer tissues showed specific overexpression of Rab22a. The overexpressed Rab22a can activate the PI3K/AKT/mTOR signaling pathway, thereby promoting EMT of thyroid cancer cells and enhancing the proliferation, migration, and invasion of thyroid cancer cells. These results suggest that Rab22a may be a potential diagnostic marker and therapeutic target for thyroid cancer.

## Figures and Tables

**Table 1:** Association between Rab22a expression and clinical parameters in 101 patients with thyroid disease.

Clinicopathological characteristics	Rab22a positive	Rab22a negative	Total	*P*
Age (Y)				
≤55	42	11	53	0.280
>55	12	6	18	
Gender				
Male	15	6	21	0.554
Female	39	11	50	
Tumor size				
≤2 cm	35	13	48	0.553
>2 cm	19	4	23	
Lymphatic metastasis				
Negative	23	8	31	0.746
Positive	31	9	40	
TNM stage				
I	45	14	59	0.925
II-IV	9	3	12	
*BRAF*				
Wild-type	27	8	35	0.854
V600E	19	5	24	
Histological type				
Papillary thyroid carcinoma	24	7	31	<0.001
Follicular carcinoma	24	9	33	<0.001
Thyroid medullary carcinoma	6	1	7	<0.001
Nodular goiter	0	30	30	

*P* <0.05 (Fisher's test).

## Data Availability

The datasets supporting the conclusions of this article are included within the article.

## Supplementary Materials

Supplementary Materials The antibodies used for western blotting in the present research were listed in Table S1. Co-IP was performed to evaluate the interaction of Rab22a with PI3K85α in TPC1 and K1 cells, as shown in Figure S1. The expression of Rab22a upon PI3K inhibitor treatment at different concentrations was analyzed by western blot in TPC1 and K1 cells.
