# Supplementary material for: Rab22a Promotes Epithelial-Mesenchymal Transition in Papillary Thyroid Carcinoma by Activating PI3K/AKT/mTOR Signaling Pathway
Source: Biomed Res Int. 2022 Jun 15;2022:1874550. doi: 10.1155/2022/1874550 (PMC9217539; doi:10.1155/2022/1874550)
Supplement: Supplementary Materials — The antibodies used for western blotting in the present research were listed in Table S1. Co-IP was performed to evaluate the interaction of Rab22a with PI3K85α in TPC1 and K1 cells, as shown in Figure S1. The expression of Rab22a upon PI3K inhibitor treatment at different concentrations was analyzed by western blot in TPC1 and K1 cells. [file 1874550.f1.zip › Table S1.docx]

| Antibody name | Source | Host | Dilution |
| --- | --- | --- | --- |
| Rab22a | ATLAS ANTIBODIES | Rabbit | 1:1000 |
| PI3K85α | Proteintech | Mouse | 1:1000 |
| p- PI3K85α | ZENBIO | Rabbit | 1:1000 |
| AKT | Proteintech | Mouse | 1:1000 |
| p-AKT(Ser473) | Proteintech | Mouse | 1:1000 |
| mTOR | Cell Signaling Technology | Rabbit | 1:1000 |
| p-mTOR(Ser2448) | Cell Signaling Technology | Rabbit | 1:1000 |
| P70S6K | Cell Signaling Technology | Rabbit | 1:1000 |
| p-P70S6K(Ser371) | Cell Signaling Technology | Rabbit | 1:1000 |
| p-P70S6K(Thr389) | Cell Signaling Technology | Rabbit | 1:1000 |
| P-4E-BP1(Thr37/46) | Cell Signaling Technology | Rabbit | 1:1000 |
| ZO-1 | Cell Signaling Technology | Rabbit | 1:1000 |
| E-cadherin | Cell Signaling Technology | Rabbit | 1:1000 |
| N-cadherin | Cell Signaling Technology | Rabbit | 1:1000 |
| Vimentin | Cell Signaling Technology | Rabbit | 1:1000 |
| Snail | Cell Signaling Technology | Rabbit | 1:1000 |
| Slug | Cell Signaling Technology | Rabbit | 1:1000 |
| GAPDH | Origene | Mouse | 1:1000 |
| β-Actin | Proteintech | Rabbit | 1:1000 |
| Anti-Rabbit | Cell Signaling Technology | Goat | 1:8000 |
| Anti-Mouse | Origene | Goat | 1:8000 |

**Table S1** Antibodies used for western blotting in the present research
